# Changing Epidemiology of Bariatric Surgery in the UK: Cohort Study Using Primary Care Electronic Health Records

**DOI:** 10.1007/s11695-015-2032-9

**Published:** 2016-01-12

**Authors:** Helen P. Booth, Omar Khan, Alison Fildes, A. Toby Prevost, Marcus Reddy, Judith Charlton, Martin C. Gulliford, for the King’s Bariatric Surgery Study Group

**Affiliations:** 1 Department of Primary Care and Public Health Sciences, King’s College London, 6th Floor, Capital House, 42 Weston St, London, SE1 3QD UK; 2 Department of Surgery, St George’s Hospital, Blackshaw Rd, London, SW17 0QT UK; 3 NIHR Biomedical Research Centre at Guy’s and St Thomas’ NHS Foundation Trust, Great Maze Pond, London, SE1 9RT UK

**Keywords:** Bariatric surgery, Gastric bypass, Gastric banding, Sleeve gastrectomy, Obesity, Body weight, Diabetes mellitus, Primary care

## Abstract

**Background:**

This study aimed to use primary care electronic health records to evaluate the epidemiology of bariatric surgery in the UK.

**Methods:**

A cohort comprising all obese patients with a bariatric surgical procedure was drawn from the Clinical Practice Research Datalink (CPRD). Rates of bariatric surgery were estimated using the registered CPRD population as denominator.

**Results:**

There were 3039 adult obese patients with first bariatric surgery procedures between 2002 and 2014, including laparoscopic adjustable gastric banding (LAGB), 1297; gastric bypass (GBP), 1265; and sleeve gastrectomy (SG), 477. Annual procedures increased from one in 2002 to a maximum of 525 in 2010. Intervention rates were greatest among those aged 35–54, with a peak of 37 procedures per 100,000 population per year in women and 10 per 100,000 per year in men. The mean age and body mass index of participants increased, as did the proportion of men and proportion with diabetes. Between 2002 and 2006, LAGB accounted for >90 % of procedures; in 2014, GBP accounted for 52 % and SG 26 %. Among patients initially receiving LAGB, the rate of band removal was 1.6 (95 % confidence interval 1.3–2.0) per 100 patient years; the rate of a second procedure of a different type was 1.2 (0.9–1.5) per 100 patient years.

**Conclusions:**

Numbers of bariatric surgical procedures have increased with increasing use of GBP and SG. Rates of bariatric surgery per 100,000 population remain low and provide evidence of limited access to bariatric surgical procedures in relation to need.

## Introduction

Recent increases in obesity have been associated with a disproportionate increase in the numbers of people affected by severe and morbid obesity [1, 2]. Bariatric surgery is now recognised as an effective treatment option for patients with morbid obesity or for those with severe obesity and comorbidities that do not respond to medical therapy [3]. Bariatric surgery is associated with reduced incidence of new diabetes [4, 5], remission of current diabetes [6], reduced prescription drug utilisation, improved quality of life [7] and reduced mortality [8]. Nevertheless, the role of surgery as a treatment for obesity in publicly funded health systems remains controversial. Improved epidemiological data are therefore required in order to inform policy development, service commissioning and clinical decision-making. The present study provides a population-based investigation of the changing epidemiology of bariatric surgery in the UK, using primary care electronic health records (EHRs). The study aimed to estimate utilisation rates for different procedures, changes in case-mix over time and the rate of re-operation.

## Methods

### Data Source

The UK Clinical Practice Research Datalink (CPRD) provided the source of electronic health records for this study. The CPRD presently holds more than 80 million person years of research-quality data from 1990 onwards from more than 600 family practices. Data held within CPRD are considered to be broadly representative of the UK population [9, 10]. Scientific and ethical approval of the protocol for the study was given by the CPRD Independent Scientific Advisory Committee (ISAC 13_089).

### Participants

The study sample comprised a cohort of adult obese patients with first bariatric surgery procedures performed, including all participants in CPRD with laparoscopic adjustable gastric banding (LAGB), gastric bypass (GBP) or sleeve gastrectomy (SG) recorded up to 30 April 2014. The date of the first procedure was taken as the index date. Participants were excluded if they did not have a BMI record for obesity (BMI ≥ 30 kg/m^2^) prior to surgery or if they were less than 20 years of age. Procedures recorded within 1 year of the participant start date in CPRD were also excluded because such records might refer to procedures performed before the patient’s registration at a CPRD practice. Participants with more than one procedure type coded on the index date or gastric band removal recorded prior to the index date were also excluded.

### Reliability Study

A sample of 102 participants was selected for a reliability study in which EHR data were compared with general practitioner-reported information. The sample selected for study included approximately equal numbers of participants with EHR records for LAGB, SG or GBP. LAGB patients who had records of gastric band removal and GBP and SG patients who had repeat procedures were oversampled. The general practitioner (GP) for each patient was sent a questionnaire which included items concerning whether the patient had bariatric surgery, the date of surgery, type of procedure, complications experienced, gastric band removal, operation reversal and repeat procedures.

### Main Measures and Analysis

The rate of utilisation of bariatric surgical procedures was estimated for men and women and for three age groups: 20 to 34, 35 to 54 and 55 to 84 years. The denominator was person years at risk for the general population registered in CPRD. The participants were classified according to the procedure recorded on the index date into LAGB, SG or GBP. Utilisation of the three procedures, as a proportion of all bariatric surgical procedures, was evaluated from 2002 to 2014. Bariatric surgical codes recorded after the index date were evaluated, and participants were classified as having a second operation if a procedure of a different type was recorded more than 30 days after the index date. In participants whose initial procedure was LAGB, we evaluated for whether a code for removal of the gastric band was recorded. The occurrence of repeat operations and band removal was evaluated in a time-to-event framework, and annual incidence rates were estimated. Records of body weight, height and BMI were identified in order to estimate changes in body weight following the index date.

## Results

The total registered population of CPRD was 4.1 million in 2002, increasing to 4.8 million from 2007 to 2010, before declining to 3.9 million in 2014. There were 4793 participants with bariatric surgery recorded; 1324 participants with bariatric surgery first recorded less than 1 year after the start of the patient record were excluded, as were 14 participants aged less than 20 years at the index date and 401 participants with either no BMI record before surgery or BMI values less than 30 kg/m^2^ prior to surgery. Nine participants with a record of gastric band removal before the index date were also excluded. There were then 3045 patients identified as having bariatric surgery for obesity. Six participants with more than one type of procedure recorded on the index date were excluded, leaving 3039 for further analysis.

### Reliability Study

Completed questionnaires were received for 78 patients (Table 1). All 78 responses confirmed that bariatric surgery had been performed on the date indicated in electronic health record data. The type of bariatric surgical procedure was confirmed for all 30 (100 %) patients recorded with LAGB, for 24 out of 25 (96 %) recorded with SG and for 19 out of 23 (83 %) recorded with GBP. Gastric band removal was confirmed for 27 out of 30 (90 %) cases. Among nine patients with second procedures recorded in EHRs following GBP or SG, six were confirmed in GP questionnaire responses. Problems relating to gastric bands were reported in 6 out of 32 cases, but high rates of complications in this group may be expected as patients requiring further procedures were oversampled. The funding source for surgery was private in 32 (41 %), National Health Service in 42 (54 %) and unspecified in 4.

**Table 1:** Reliability study of bariatric surgery comparing primary care electronic health records (EHR) with responses from general practitioner questionnaires. Figures are frequencies except where indicated

	EHR	GP questionnaire	Percent agreement (95 % CI)
Bariatric surgery performed	78	78	100 (–)
Surgery type			
Adjustable gastric banding	30	30	100 (–)
Gastric bypass (GBP)	23	19	83 (61, 95)
Sleeve gastrectomy (SG)	25	24	96 (80, 100)
Gastric band removal	30	27	90 (73, 98)
Procedure secondary to GBP	3	1	33 (1, 91)
Procedure secondary to SG	6	5	83 (36, 100)
	Difference in date (days, median, IQR)		
Date of primary bariatric surgical procedure	0 (0, 0)		

*EHR* electronic health record, *GP* general practitioner, *IQR* interquartile range

### Utilisation of Bariatric Surgical Procedures

**Fig. 1:**
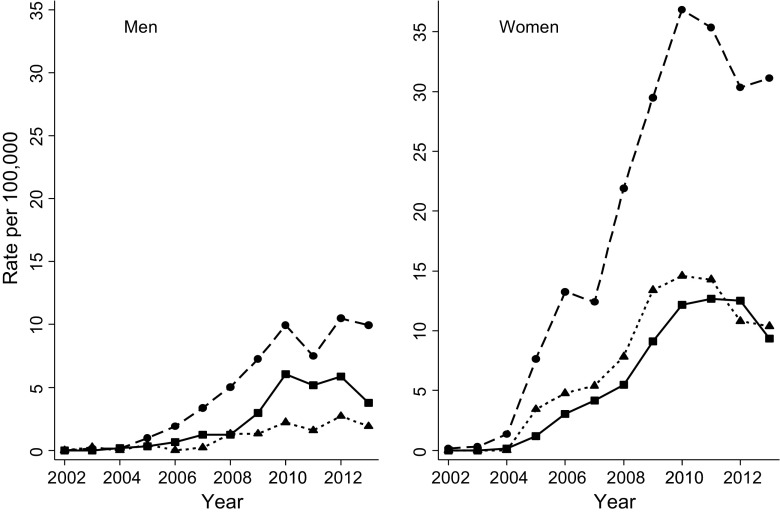
Rates of first bariatric surgery procedures in a large primary care population. *Dotted lines* 20 to 34 years, *dashed lines* 35 to 54 years, *solid lines* 55 to 84 years

**Fig. 2:**
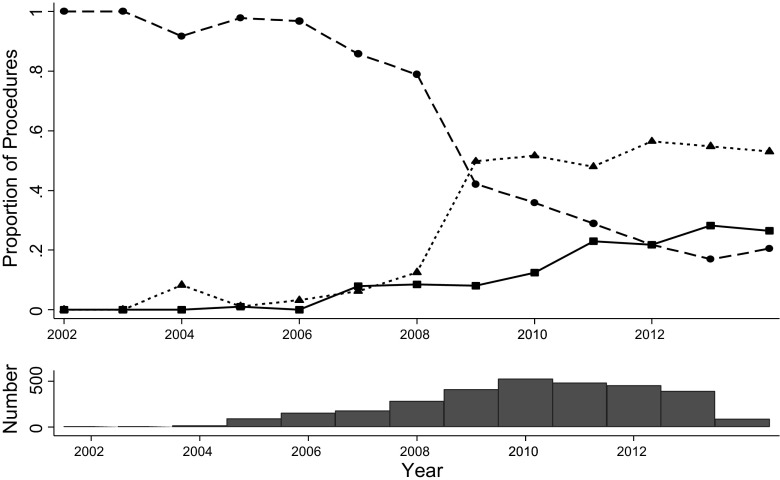
Trends in the utilisation of different bariatric surgical procedures from 2002 to April 2014 (*upper panel*) and total number of procedures per year (*lower panel*). *Dashed line* LAGB, *dotted line* gastric bypass, *solid line* sleeve gastrectomy

The number of procedures recorded increased over time (Table 2). The rate of surgery was highest in men and women aged 35 to 54 years. Rates of bariatric surgical procedures by age group and gender are presented in Fig. 1. Rates of bariatric surgery were greatest for women in 2010 at 37 per 100,000 population per year and in 2012 for men at 10 per 100,000. Disparity between genders was greatest in the youngest patients, aged 20 to 34, with peak rates of 15 per 100,000 per year in women and 3 per 100,000 per year in men. LAGB was the most frequent procedure, accounting for 1297 (43 %) cases, followed by GBP in 1265 (42 %) and SG in 477 (16 %). LAGB accounted for 97 % of 104 procedures performed from 2002 to 2005. The use of GBP and SG increased over time while LAGB declined (Fig. 2). During 2012 to 2014, GBP accounted for 55 % of procedures while SG accounted for 25 % and LAGB, 20 % (Table 2).

**Table 2:** Characteristics of patients receiving first bariatric surgery procedures from 2002 to 2014. Figures are frequencies (column percent)

	2002–2005	2006–2008	2009–2011	2012–2014	
Number of procedures	104	607	1406	922	
Type of procedure					<0.001
Gastric banding	101 (97)	518 (85)	497 (35)	181 (20)	
Gastric bypass	2 (2)	51 (8)	701 (50)	511 (55)	
Sleeve gastrectomy	1 (1)	38 (6)	208 (15)	230 (25)	
Age at procedure (median, IQR)	43.4 (8.6)	44.4 (10.0)	46.1 (10.4)	46.8 (10.0)	<0.001
Female	89 (86)	504 (83)	1118 (80)	691 (75)	<0.001
Body mass index (BMI, kg/m^2^, mean SD)	40.6 (7.1)	42.7 (8.3)	44.2 (8.2)	44.8 (8.3)	<0.001
BMI category (kg/m^2^)					
30–34.9	29 (28)	108 (18)	162 (12)	95 (10)	<0.001
35.0–39.9	24 (23)	161 (27)	301 (21)	189 (21)	
≥40	51 (49)	338 (56)	943 (67)	638 (69)	
Diabetes	20 (19)	124 (20)	428 (30)	302 (33)	<0.001
Depression	61 (59)	320 (53)	762 (54)	540 (59)	0.148
Current smoking	20 (19)	104 (17)	231 (16)	146 (16)	0.323
Antihypertensive drugs	42 (40)	278 (46)	728 (52)	509 (55)	<0.001
Statins	20 (19)	123 (20)	418 (30)	301 (33)	<0.001

### Changes in Case-Mix

Patient characteristics at the index date are presented in Table 3. The mean age at operation increased from 43.4 to 46.8 years during the study (*P* < 0.001), and the proportion of women declined from 86 to 75 % (*P* < 0.001). The mean recorded BMI increased from 40.6 to 44.8 kg/m^2^ (*P* < 0.001). The proportion of participants with diabetes increased from 19 to 33 %, while the proportion of patients prescribed antihypertensive drugs and statins also increased (all *P* < 0.001). More than half of all participants had depression recorded at some time before the procedure. As a consequence of these trends, there were important differences in case-mix for patients undergoing LAGB as compared to GBP and SG (Table 3). LAGB patients were generally operated on in an earlier period, were younger, were more often female, were less obese and were less likely to have diabetes, hypertension or hypercholesterolaemia.

**Table 3:** Variables associated with use of gastric bypass or sleeve gastrectomy rather than gastric banding

	LAGB (1297)	Gastric bypass/sleeve (1742)	Odds ratio (95 % CI)	*P* value
Period of procedure				
2002–2005	101 (8)	3 (0)	0.21 (0.06 to 0.68)	0.009
2006–2008	518 (40)	89 (5)	Ref.	
2009–2011	497 (38)	909 (52)	11.7 (8.61 to 15.9)	<0.001
2012–2014	181 (14)	741 (43)	26.0 (18.7 to 36.3)	<0.001
Age (median, IQR)	44.3 (10.0)	47.1 (10.2)	1.017 (1.007 to 1.027)	0.001
Female	1103 (85)	1299 (75)	0.66 (0.53 to 0.82)	<0.001
Body mass index (BMI, kg/m^2^, mean SD)	41.3 (7.3)	46.0 (8.4)	1.08 (1.07 to 1.09)	<0.001
Diabetes	244 (19)	630 (36)	1.49 (1.18 to 1.89)	0.001
Depression	715 (55)	968 (56)	1.03 (0.86 to 1.24)	0.754
Currently smoking	229 (18)	272 (16)	0.95 (0.75 to 1.21)	0.663
Antihypertensive	572 (44)	985 (57)	1.00 (0.83 to 1.22)	0.960
Statins	255 (20)	607 (35)	1.20 (0.91 to 1.59)	0.198

### Secondary Procedures

There were three deaths within 30 days of the date of the initial procedure. Rates of band removal and re-operation following LAGB are presented in Table 4. The most common procedure was removal of a gastric band, found in 82 (6.3 %) cases. This was equivalent to a rate of 1.6 (95 % confidence interval 1.3 to 2.0) per 100 person years, and the median time between gastric band insertion and removal was 144 weeks (IQR 69 to 203). There were 60 (4.6 %) LAGB patients who had a subsequent medical code recorded indicating a GBP or SG procedure, with a rate of 1.2 (0.9 to 1.5) per 100 patient years. There were 10 patients who received SG, who later had codes for gastric bypass recorded, and six patients with GBP, who later had codes for LAGB (4) or SG (2) recorded.

**Table 4:** Re-operation using a second type of procedure and band removal following initial bariatric surgical procedures

First procedure	Subsequent procedure	Freq. (%)	Median interval (IQR, weeks)	Rate per 100 patient years (95 % confidence interval)
LAGB (1297)	Band removed	82 (6.3)	144 (69 to 203)	1.6 (1.3 to 2.0)
	Subsequent bypass or sleeve	60 (4.6)	108 (58 to 200)	1.2 (0.9 to 1.5)

*IQR* interquartile range, *LAGB* laparoscopic adjustable gastric banding

## Discussion

This large-scale population-based study of the utilisation of bariatric surgical procedures in the UK complements a recently published bariatric surgical registry report [11]. The rate of bariatric surgery recorded in primary care medical records increased rapidly between 2002 and 2014. Initially, laparoscopic adjustable gastric banding (LAGB) accounted for most procedures, but the use of gastric bypass, and to a lesser extent sleeve gastrectomy, has increased since 2008. There have been changes in case-mix with procedures now being performed in older patients, with greater BMI and a higher prevalence of diabetes. These results are consistent with changes in surgical practice reported internationally, though the decline in gastric banding may have been more rapid elsewhere [12]. The increase in number of bariatric surgery procedures identified in CPRD over the last 10 years is consistent with findings reported from analysis of hospital utilisation statistics [13]. The gender disparity, age profile of surgery patients and changing patterns of surgery were also comparable to the trends seen in data for hospital utilisation, bariatric surgical registry data and international surveys [11–13].

Overall rates of bariatric surgery remain extremely low. National survey data show that 1.7 % of men and 3.1 % of women in England have morbid obesity [1]. An estimated two million individuals are potentially eligible for bariatric surgery based on body weight [14]. Capacity is presently limited [15] and National Health Service policy guidance presently recommends only a gradual and limited increase in the rate of bariatric surgery [16]. Consequently, a high proportion of procedures are performed in the private sector, as evidenced in the questionnaire study, and this may lead to inequity in access to surgery [17].

Reconsideration of eligibility for bariatric surgery may require attention to metabolic parameters and comorbidity, as well as body mass index, as selection criteria [18]. In this context, the role of bariatric surgery in the prevention or resolution of morbidity is important [19] and we report elsewhere on diabetes incidence and depression diagnoses in this cohort [4, 20]. Paradoxically, although BMI remains a primary selection criterion, follow-up in primary care with respect to changes in body weight was poor with only 18, 15 and 13 % of participants having weight values recorded in the first 3 years after surgery.

Following gastric banding, gastric band removal was observed in 1.6 % of patients per year and 1.2 % per year were recorded as having a further additional procedure of gastric bypass or sleeve gastrectomy. These findings confirm in population-based data that there is significant incidence of band slippage or band intolerance requiring removal. The bariatric surgical registry recorded a much lower proportion of patients undergoing revisional bariatric surgery (0.3 %) after gastric banding [11]. This discrepancy may reflect the short period (3 years) covered by registry data, problems with data linkage occurring when re-operations are performed at different hospitals or under-reporting of re-operations and revisions.

This study had the strengths of a large nationally representative data source with extended periods of longitudinal follow-up. We acknowledge that clinical information has several limitations when used for research purposes, including missing data values due to opportunistic data collection and recording, but a reliability study suggested a high level of agreement between EHR records and GP reports for primary surgeries. This is the first large-scale study to use electronic health records for the evaluation of bariatric surgical utilisation for obesity and that demonstrates rapid increases in the use of such procedures and a move away from gastric banding towards gastric bypass and sleeve gastrectomy, with a shift in case-mix towards more severely affected patients.
